# Identification of Chemotherapy-Induced Peripheral Neuropathy—A Self-Administered Scoring System Tested in Breast Cancer Survivors: Protocol of the NEURO-BREAC Trial

**DOI:** 10.3390/jpm15110554

**Published:** 2025-11-13

**Authors:** Dirk Rades, Maria Karolin Streubel, Laura Doehring, Achim Rody, Martin Ballegaard

**Affiliations:** 1 Department of Radiation Oncology, University of Lübeck, 23562 Lübeck, Germany; 2 Department of Radiation Oncology, University Medical Center Schleswig-Holstein, Lübeck Campus, 23538 Lübeck, Germany; mariakarolin.streubel@uksh.de (M.K.S.); laura.doehring@uksh.de (L.D.); 3 Department of Obstetrics and Gynecology, University Medical Center Schleswig-Holstein, Lübeck Campus, 23538 Lübeck, Germany; achim.rody@uksh.de; 4 Department of Neurology, Zealand University Hospital, 4000 Roskilde, Denmark; mbag@regionsjaelland.dk; 5 Department of Clinical Medicine, Faculty of Health and Medical Sciences, University of Copenhagen, 2200 Copenhagen, Denmark

**Keywords:** breast cancer, early detection, neuropathy, scoring system, taxane-based chemotherapy

## Abstract

**Background/Objectives**: Many patients with breast cancer are treated with chemotherapy, including taxanes. These regimens bear a significant risk of potentially burdensome peripheral neuropathy. A scoring system supported by a neuropathy tracker, which can be self-administered by the patients, likely facilitates and speeds up the diagnosis of chemotherapy-induced peripheral neuropathy (CIPN). Before such a scoring system can be used, determination of the optimal cut-off score to discriminate between CIPN and no CIPN is necessary. The prospective NEURO-BREAC trial (NCT07148336) aims to identify the optimal cut-off score in patients treated with chemotherapy and adjuvant irradiation for breast cancer. **Methods**: The main goal of the NEURO-BREAC trial is to provide the optimal cut-off of a scoring system to discriminate between moderate to severe CIPN and no CIPN in breast cancer survivors previously treated with paclitaxel- or docetaxel-based chemotherapy and irradiation. The scores (0 to 44 points) are obtained by using a neuropathy tracker. This tracker is based on self-evaluation of symptoms and signs of CIPN by study participants. In addition, satisfaction of the patients with the scoring system is assessed. Twenty-four patients (sixteen patients with moderate to severe CIPN and eight patients without CIPN) are required for the Full Analysis Set. Assuming that about 5% of patients will not qualify for this set, 26 patients should be recruited for the NEURO-BREAC trial. The results of this trial are considered an important step for the development of a scoring system contributing to the identification of CIPN in breast cancer patients.

## 1. Introduction

In case of more advanced tumor stage and/or presence of specific risk factors, the patients with breast cancer who are assigned to local treatment, namely surgery with or without postoperative irradiation, may be candidates for preoperative or postoperative chemotherapy [1]. Typically, the chemotherapy regimens used in these situations include paclitaxel or docetaxel, which both bear a high risk of leading to chemotherapy-induced peripheral neuropathy (CIPN) [2]. Some of the commonly used regimens include also carboplatin, which can further increase the risk of CIPN [3]. CIPN can be onerous for the affected patients, particularly in the case of moderate to severe CIPN [4,5,6,7,8,9]. Moderate to severe CIPN is associated with limitations regarding instrumental and self-care activities of daily living [10].

According to the current literature, there is a lack of prophylactic treatment to avoid peripheral neuropathy [2,11,12], and options for treating this complication are quite limited. Duloxetine was demonstrated to improve neuropathy-related pain but not sensory or motor deficits [13,14,15,16,17]. Considering these limited options, early diagnosis of CIPN and subsequent adaption of the chemotherapy regimen may be helpful to prevent significant long-term CIPN [7,8,9,18,19,20]. Moreover, close monitoring during and following chemotherapy is important to adapt the regimen immediately after initial symptoms of CIPN. The frequently used UENS needs to be performed by specially trained health care professionals and requires a 1¾ inch safety pin (pinprick sensation) and a 128 Hz tuning fork (vibration) [21]. For the TNS, which is also performed by trained health care professionals, a tuning fork, a safety pin, and a reflex hammer are needed [22,23,24,25]. Therefore, an additional tool that can be easily administered by the patients at home appears desirable. One of the first steps in this context would be the development of a suitable scoring system that can be used by the patients without the assistance of health care professionals. Such a scoring system has already been created by members of our research group. This system considers number and seriousness of symptoms identified by the patients with the help of a neuropathy tracker [26]. The primary aim of the current NEURO-BREAC trial is to determine the optimal score to discriminate between moderate to severe and no CIPN.

## 2. Experimental Design and Materials

### 2.1. Aims of the Trial

The primary aim of the NEURO-BREAC trial is to determine the optimal cut-off score of a scoring system to discriminate between moderate to severe CIPN and no CIPN in breast cancer survivors previously treated with chemotherapy containing a taxane and adjuvant radiotherapy. The scores that range between 0 and 44 points are obtained by using a neuropathy tracker [26]. This tracker is based on self-evaluation of signs of CIPN by study participants. The receiver operating characteristic (ROC) curve is applied to demonstrate the relation between sensitivity and specificity for each cut-off score and determine the optimal score for identification of moderate to severe CIPN. In addition, satisfaction of the study participants with the scoring system is evaluated (secondary aim).

### 2.2. Design and Duration of the Trial

The prospective NEURO-BREAC trial is a monocentric study, which is performed to identify the optimal cut-off score of a scoring system to discriminate between moderate to severe CIPN and no CIPN in breast cancer survivors who previously received taxane-based chemotherapy and radiotherapy. Completion of the inclusion of the planned 26 patients is estimated to take 9 months. Time between recruitment and completion of the study procedures will be up to 1 month. Thus, the NEURO-BREAC trial will be finalized after about 10 months.

### 2.3. Eligibility Criteria

The NEURO-BREAC trial will be performed in patients previously treated with taxane-based chemotherapy and subsequent adjuvant radiotherapy for breast cancer. Patients are required to have either no CIPN or moderate to severe CIPN. Moderate CIPN is defined as limiting instrumental activities of daily living (e.g., shopping, housekeeping, using the telephone) and severe CIPN as limiting self-care activities of daily living (e.g., bathing, dressing) [10]. The study participants must be adequately informed about the trial. They may only be included after pre-therapy clarification and on fulfillment of all inclusion criteria and on non-fulfillment of all exclusion criteria shown in Table 1.

### 2.4. Termination of the NEURO-BREAC Trial

The trial ends after completion of study procedures (self-assessment by the patients regarding symptoms and signs of CIPN, completion of the questionnaire regarding satisfaction with the scoring system) by the last study participant. The trial may be stopped prematurely by the principal investigator if necessary. Possible reasons for premature termination include the situation that new data from the literature make the current trial redundant or the fact that the trial cannot be performed as planned in daily practice. Moreover, a study participant may terminate participation in the trial at any time.

### 2.5. Sample Size Calculation

The primary aim of the NEURO-BREAC trial is to identify the optimal cut-off score of a scoring system to discriminate between moderate to severe CIPN and no CIPN in breast cancer survivors. For assessment of the discriminative power of the scoring system, the area under the receiver operating characteristic (ROC) curve (AUC) is calculated. The rationale and procedure of the sample site calculation have already been used in a previous trial [27]. The calculation of the sample size considered six assumptions (a. to f.):

(a.) The two-sided significance level is set to 10%; (b.) an AUC of 0.95 is assumed since this is decided to be an excellent diagnostic accuracy for the scoring system worth to be considered for future routine use; (c.) the pre-specified lower bound of the confidence interval is assumed to be 0.7; (d.) the assurance probability is set to 80%; (e.) the ratio of standard deviations in patients with no CIPN and patients with moderate to severe CIPN is assumed to be 0.5; (f.) approximately 33% of the patients to be included in this study will have no CIPN (according to standard physical examination and medical history), whereas the other 67% will be clinically diagnosed with moderate to severe CIPN, i.e., ratio between negative and positive cases is approximately 0.5. Thus, 24 patients (16 with moderate to severe CIPN and 8 without moderate to severe CIPN) are needed within the Full Analysis Set, which includes all patients who completed the self-assessment of CIPN. As this is a monocentric study, we maintain close oversight of all study participants, including standardized patient information and consent procedures. Based on this controlled setting, we do not anticipate significant issues with compliance or reporting bias. Baseline characteristics will be collected and reported. We acknowledge that, due to the limited sample size and single-center design, the risk of selection bias—in the sense that the study population may not be fully representative of the broader target population—cannot be entirely excluded. Importantly, this study is not intended to provide a definitive validation of the scoring system but to explore its potential usefulness in a prospective setting and to contribute to the design of larger, multicenter studies in the future.

## 3. Treatment and Study Procedures

### 3.1. Assessments and Procedures

Data will be assessed at the University of Lübeck, Germany. At the beginning of the study, medical history (including smoking history), concomitant diseases (including diabetes, hypertension, autoimmune disease, and cardiovascular disease), concomitant medication (including beta-blockers), physical examination, demographics (age, date of birth, gender), body mass index (body height and body weight), Karnofsky performance score, TNM-stage, histology, surgery, radiotherapy, timing of chemotherapy (neoadjuvant, adjuvant), and the previously administered chemotherapy regimen will be recorded (Table 2). Taxane-based chemotherapy regimens allowed in the NEURO-BREAC trial, types of surgery, and details of adjuvant radiotherapy were previously reported [28,29].

During the NEURO-BREAC trial, symptoms and signs of CIPN assessed by study participants, satisfaction of the study participants with the scoring system, symptoms and signs of CIPN assessed by health care professionals, and adverse events will be assessed (Table 2). The patients will be asked to complete the self-evaluation of symptoms and signs of neuropathy using the neuropathy tracker [26]. They will be handed a mobile with the software pre-installed. After a training session and detailed instructions from health care professionals, the study participants will perform the procedure without supervision in a separate room but with the possibility of calling for help. The neuropathy tracker questions symptoms quality, severity, and distribution, and guides the user through a systematic evaluation of pinprick from a needle and vibration from the mobile on successive levels from the toes to the knee on both legs. Finally, the extension force of both big toes will be assessed by the participant. The self-examination is based on the structure of the Utah Early Neuropathy Score [21]. After completion of the self-assessment of CIPN, the patients will be asked to complete a questionnaire (based on and modified according to [30,31]), regarding their satisfaction with the scoring system. If the dissatisfaction rate is greater than 20%, the system needs to be adapted prior to future use. If the dissatisfaction rate is greater than 40%, the system will be regarded not useful to help identify moderate to severe CIPN in breast cancer survivors.

The assessment of symptoms and signs of CIPN by health care professionals is performed using the Utah Early Neuropathy Scale (UENS) and the Total Neuropathy Score (TNS). The UENS [21] requires a number 2 (1¾ inch) safety pin and a 128 Hz tuning fork. The clinical part of the TNS [22,23,24,25] includes severity of sensory and motor symptoms, number of autonomic symptoms, reduction in pin sensitivity, reduction in vibration sensibility, weakness, and reduction in reflexes. Depending on severity, 0 points (no symptoms, normal findings) to 4 points (highest degree of symptoms or signs) are assigned to each of these seven symptoms. Scores for individual patients range between 0 and 28 points. According to Velasco et al. [32], severity of CIPN could be graded as follows: no PNP = 0 points, grade 1 (representing mild PNP) = 1–7 points, grade 2 (representing moderate PNP) = 8–14 points, grade 3 (representing severe PNP) = 15–21 points, and grade 4 (representing very severe PNP) = 21–28 points. Thus, moderate to severe PNP is represented by scores > 7 points. Adverse events other than CIPN will also be assessed [10].

### 3.2. Statistical Analyses

All data recorded in the case report forms describing the study population, rates of moderate to severe CIPN, and safety will be analyzed descriptively. Categorical data will be presented in contingency tables with frequencies and percentages. Continuous data will be summarized including frequency (n), median, quartiles, mean, standard deviation, minimum, and maximum. Number of patients with and types of protocol deviations will be provided. The data analysis will be performed according to a statistical analysis plan.

The primary aim of the study is to identify the optimal cut-off score of a scoring system to discriminate between moderate to severe CIPN and no CIPN in breast cancer survivors. The evaluation will be performed in study participants, who based on standard physical examination and medical history were previously classified to have either no or moderate to severe CIPN. Sensitivity and specificity will be estimated for every possible cut-off value of the scoring system. The ROC curve is used to show the relation between sensitivity and specificity. The area under the curve (AUC) summarizes the location of the ROC curve. If the AUC is 1.0, the cut-off score is perfect in the differentiation between subjects with moderate to severe CIPN and with no CIPN. An AUC of 0.5 means that the scoring system is performing no better than chance. A score leading to an AUC of ≤0.7 will be rated not useful. Non-parametric methods for AUC estimation and testing will be used [33]. The SAS 9.4 (SAS Institute Inc.) LOGISTIC procedure with the ROCCONTRAST statement will be used to estimate the AUC to provide the *p*-value for the test described above. A significance level of two-sided 10% is pre-specified. If statistical significance of the AUC is achieved, the most informative score to predict moderate to severe CIPN will be determined. A cut-off score with a sensitivity of ≥90% and a specificity of ≥80% was defined as optimal. Moreover, the Youden index (sensitivity + specificity − 1) will be calculated to propose an optimal cut-off score. The derived cut-off value should be considered as a preliminary suggestion which has to be validated in subsequent studies. As a further sensitivity analysis, the relationship between tertiles of the scores and the incidence of moderate to severe CIPN will be evaluated using the Jonckheere–Terpstra test. It tests the global null hypothesis that the distribution of the response variable does not differ among the tertiles. It is created to detect alternatives of ordered differences, meaning that the incidence of moderate to severe CIPN increases with the tertiles of score values. We applied the Jonckheere–Terpstra test to specifically assess whether there is a monotonic trend in the frequency of moderate to severe CIPN across increasing score levels. This reflects our a priori scientific hypothesis: higher scores are associated with a higher probability of clinically relevant neuropathy. If no such monotonic relationship exists, the practical utility of the scoring system would be questionable. Therefore, the Jonckheere–Terpstra test was chosen deliberately to evaluate this specific assumption. We acknowledge that the test is not designed to detect non-monotonic patterns. Such patterns would not support the intended use of the score in clinical practice and are thus not the focus of our analysis. Importantly, the Jonckheere–Terpstra test was used as part of a sensitivity analysis, serving as an additional, supportive approach to confirm the robustness of our findings. Importantly, our scoring system is intended to support a binary decision (i.e., presence or absence of CIPN) based on a defined threshold. It does not provide probabilistic risk estimates. Therefore, calibration metrics such as calibration plots or the Brier score, which are essential for evaluating probabilistic models, are not applicable in this context.

In addition, the impact of potential risk factors including smoking history, diabetes, hypertension, autoimmune disease, significant cardiovascular disease, treatment with beta-blockers, age, gender, body mass index, Karnofsky performance score, tumor stage, histology, type of surgery, timing of chemotherapy (neoadjuvant, adjuvant), and type of chemotherapy on the occurrence of moderate to severe CIPN will be evaluated applying Chi-square or Fisher’s exact tests.

Satisfaction with the scoring system will be assessed after completion of the self-assessment of symptoms and signs of CIPN by the study participants using the neuropathy tracker. The statistical analysis will provide the respective proportions (please, refer also to 3.1).

### 3.3. Data Management and Data Protection

Data management and data protection represent standard procedures at our institution and have been previously reported in detail [27,33].

### 3.4. Dissemination of Results and Publication Policy

Dissemination of results and publication policy also follow standard procedures and have been previously reported [27,33]. Conclusions drawn from the NEURO-BREAC trial need to be approved by an external professional statistician. The acronym NEURO-BREAC will be used for publications.

## 4. Expected Results and Discussion

Breast cancer patients treated with taxane-based chemotherapy have a considerable risk to experience CIPN [2,20]. Since moderate to severe CIPN can significantly impair the patient’s instrumental and self-care activities of daily living, it is important to avoid this complication. This conclusion is further supported by the fact that the options for the treatment of CIPN are quite limited. Only pain as a symptom of CIPN can be relieved by duloxetine, which inhibits the uptake of serotonin and nor-epinephrine [13,14,15,16,17]. Thus, it appears important to detect CIPN and adapt the systemic treatment as soon as possible [7,8,9,18,19,20]. Appropriate monitoring of patients receiving paclitaxel or docetaxel should be performed both during and following their treatment. To facilitate the monitoring, a tool suitable for self-administration by the patients at home would be very helpful, since the commonly used objective tools, namely the UENS and the TNS, require special equipment, including a 1¾ inch safety pin, a 128 Hz tuning fork, and a reflex hammer [21,22,23,24,25]. This led to the idea of creating a neuropathy tracker, a smartphone-based tool enabling the patients to record symptoms and findings from self-examination in a structured neurological assessment [26]. The performance in distal symmetrical peripheral neuropathy is under publication and a study in small fiber neuropathy is ongoing. The tracker can also be used to support a new scoring system based on self-assessment of symptoms and signs of CIPN by the patients. This scoring system considers general symptoms of CIPN, pinprick sensation, hyperalgesia/allodynia, large fiber sensation, and strength. Scoring points are assigned to each of these neurological aspects, resulting in total patient scores that range between 0 and 44 points [26]. However, it is not yet known which is the optimal cut-off patient score to detect mild or moderate to severe CIPN. The aim of the present NEURO-BREAC trial represents the first step in this context, namely the definition of the optimal cut-off score for discrimination between moderate to severe CIPN and no CIPN. It is expected that the optimal cut-off score will be identified. The results of the NEURO-BREAC trial will contribute to the proper design of a phase 2 trial that evaluates the role of the scoring system supported by the neuropathy tracker in the detection of mild CIPN. However, since the study population consists of breast cancer survivors who have undergone taxane-based chemotherapy followed by radiotherapy, the scoring system may not be applicable to other clinical contexts (different chemotherapy regimens, other concurrent treatments, other demographic or disease groups). Positive and negative predictive values are not planned to be calculated, since they reflect the clinical utility of a test in a specific population and are strongly influenced by disease prevalence. In a small, single-center study, the prevalence of moderate to severe CIPN cannot be estimated without bias, and stratified analyses by baseline risk factors are not feasible. Moreover, due to the limited sample size, subgroup analyses or stratified evaluations of the diagnostic performance of the scoring system (e.g., sensitivity or specificity within subgroups) appeared not reasonable. The statistical power for detecting heterogeneity in such analyses would be insufficient in this setting. The current study is designed to provide initial prospective evidence on the overall performance of the scoring system. If the results are promising, they will serve as a basis for larger, adequately powered studies, in which subgroup analyses and multivariable adjustments can be meaningfully conducted. Thus, an additional prospective trial with a larger sample size will be required in the future.

## Figures and Tables

**Table 1:** Criteria for inclusion and exclusion of the NEURO-BREAC trial.

Criteria for inclusion Histologically proven breast cancer Previous treatment with taxane-based chemotherapy followed by adjuvant radiotherapy Moderate to severe or no CIPN according to Utah Early Neuropathy Scale and Total Neuropathy Score Female gender Age ≥ 18 years Written informed consent Capacity of the patient to consent
Criteria for exclusion Disease-related skin disorders of the lower extremities (e.g., related to infections, bullous dermatoses, dermatitis, papulo-squamous skin disorders, or urticaria/erythema) Pregnancy, lactation Expected non-compliance

**Table 2:** Timeline of enrolment, interventions, and assessments.

	Screening	Baseline	Day of the Assessment of Peripheral Neuropathy (End of Study)
Demographics	X		
Medical history and concomitant diseases		X	X
Concomitant medication		X	X
Chemotherapy details	X		
Radiotherapy	X		
Physical examination		X	X
Body mass index		X	
Performance status		X	
Pathology and tumor stage		X	
Upfront surgery		X	
Study related procedures			
Informed consent	X		
Inclusion criteria	X		
Exclusion criteria	X		
Scoring system (self-assessment by study participants)			X
Satisfaction with the scoring system			X
Utah Early Neuropathy Scale (assessed by health care professionals)			X
Total Neuropathy Score (assessed by health care professionals)			X

## Data Availability

No new data were created or analyzed in this study. Data sharing is not applicable to this article.
